# OPCAB in the minimally invasive era: impact on wound complications—a comparative study with on-pump CABG

**DOI:** 10.3389/fcvm.2026.1823140

**Published:** 2026-04-29

**Authors:** Ken Nakamura, Kentaro Akabane, Shusuke Arai, Ryota Katsura, Miku Konaka, Jun Hayashi, Eiichi Ohba, Cholsu Kim, Hideaki Uchino, Takao Shimanuki, Tetsuro Uchida

**Affiliations:** 1 Division of Cardiovascular Surgery, Nihonkai General Hospital, Sakata, Japan; 2 Second Department of Surgery, Yamagata University Faculty of Medicine, Yamagata, Japan

**Keywords:** coronary artery bypass grafting, endoscopic vein harvesting, major adverse cardiac and cerebrovascular events, off-pump coronary artery bypass, on-pump coronary artery bypass, saphenous vein graft, wound complications

## Abstract

**Background:**

The impact of off-pump coronary artery bypass grafting (OPCAB) on great saphenous vein (GSV) harvest-site wound complications and graft integrity remains uncertain in the contemporary era. We compared harvest-site morbidity, early angiographic outcomes, and long-term clinical events between OPCAB and conventional on-pump coronary artery bypass grafting (CABG).

**Methods:**

This single-center retrospective study included 394 patients who underwent isolated CABG with available pre- and postoperative coronary angiography between 2005 and 2017. Propensity score matching according to pump usage yielded 157 matched pairs [on-pump CABG, *n* = 157; OPCAB, *n* = 157]. The primary endpoint was GSV harvest-site wound complications. Secondary endpoints included early graft occlusion or saphenous vein graft (SVG) stenosis, reintervention, perioperative outcomes, and major adverse cardiac and cerebrovascular events (MACCE).

**Results:**

After matching, baseline characteristics were comparable, although the on-pump group had more distal anastomoses. Leg wound complications were rare and similar between groups (1.3% vs. 0.6%, *p* = 1.00). Early graft occlusion (9.6% vs. 10.3%, *p* = 0.553) and SVG occlusion (5.7% vs. 4.5%, *p* = 0.798) did not differ significantly. SVG stenosis severity and reintervention rates were comparable. OPCAB was associated with shorter operative time, fewer red blood cell transfusions (49.7% vs. 64.3%, *p* = 0.012), shorter intensive care unit stay, and reduced hospital stay. Thirty-day mortality and 12-month MACCE rates (12.1% in both groups) were similar. Long-term MACCE-free survival up to 10 years showed no significant difference.

**Conclusion:**

OPCAB did not reduce GSV harvest-site complications compared with on-pump CABG. Early graft integrity and long-term clinical outcomes were comparable, although OPCAB improved perioperative resource utilization.

## Introduction

Coronary artery bypass grafting (CABG) remains the standard revascularization strategy for patients with complex multivessel or left main coronary artery disease, particularly in those with diabetes mellitus and impaired ventricular function (1). Conventional on-pump CABG using cardiopulmonary bypass (CPB) and cardioplegic arrest has been the established technique for decades; however, off-pump coronary artery bypass grafting (OPCAB) was developed to avoid CPB-related systemic inflammatory response, aortic manipulation, and organ dysfunction (2).

Several large randomized controlled trials have compared OPCAB with on-pump CABG. The ROOBY trial reported inferior graft patency and worse long-term outcomes with OPCAB (3, 4), whereas the CORONARY and GOPCABE trials demonstrated comparable rates of death, myocardial infarction, stroke, and renal failure between the two strategies at mid- and long-term follow-up (2, 5). Meta-analyses have further suggested that although OPCAB may reduce early complications such as atrial fibrillation or transfusion requirements, concerns remain regarding completeness of revascularization and saphenous vein graft (SVG) durability (6, 7). Thus, the overall clinical advantage of OPCAB continues to be debated.

In the era of minimally invasive cardiac surgery, attention has expanded beyond pump avoidance to include reduction of surgical trauma and wound-related morbidity. Harvest-site complications after great saphenous vein (GSV) procurement—including leg wound infection, dehiscence, lymphatic leakage, and delayed healing—remain clinically significant, particularly in patients with diabetes mellitus, obesity, and peripheral arterial disease (5, 8). Endoscopic vein harvesting (EVH) has been shown to reduce wound complications compared with open techniques (9, 10), although early concerns were raised regarding possible adverse effects on graft patency (11, 12). Despite these advances, limited data exist regarding whether the primary revascularization strategy (OPCAB vs. on-pump CABG) independently influences GSV harvest-site morbidity.

Furthermore, objective angiographic assessment of early graft quality is not routinely incorporated into comparative studies of surgical technique. Early graft failure and SVG stenosis detected by postoperative coronary angiography (CAG) may provide mechanistic insight into long-term outcomes (13). However, few single-center propensity-matched analyses have simultaneously evaluated harvest-site wound complications, angiographic graft integrity, and long-term major adverse cardiac and cerebrovascular events (MACCE) in the contemporary era.

At our institution, OPCAB was initially adopted as the predominant strategy; over time, on-pump CABG (including arrested-heart procedures) was increasingly utilized to enhance procedural standardization and safety. This institutional transition provides a unique opportunity to compare these approaches within a relatively homogeneous perioperative management framework.

Therefore, the present study aimed to evaluate whether OPCAB confers an advantage in reducing GSV harvest-site wound complications compared with on-pump CABG, while also assessing early graft quality by postoperative CAG and long-term clinical outcomes including MACCE.

## Patients and methods

This was a single-center retrospective observational study. Between January 2005 and December 2017, 394 consecutive patients underwent isolated CABG at our institution. Patients were eligible for inclusion if both preoperative and postoperative CAG were available and if clinical follow-up data were accessible through at least the first postoperative outpatient visit. Patients were excluded if postoperative CAG was not available, if CABG was performed concomitantly with another major cardiac surgical procedure, or if CABG was performed as an unplanned intraoperative adjunct to another primary procedure. To minimize selection bias, propensity score matching was performed according to pump usage (on-pump CABG vs. off-pump CABG [OPCAB]). After matching, 157 patients were included in each group (On-pump group, *n* = 157; OPCAB group, *n* = 157).

### Surgical strategy

The choice of surgical technique (on-pump vs. off-pump) was determined by the attending surgeon based on institutional practice and patient characteristics.

On-pump CABG was performed using standard cardiopulmonary bypass with ascending aortic cannulation and right atrial drainage. Myocardial protection was achieved using cardioplegic arrest according to surgeon preference.

OPCAB was performed using cardiac stabilization devices and intracoronary shunts when necessary. Aortic manipulation was minimized whenever feasible.

The left internal thoracic artery (LITA) was preferentially used for the left anterior descending artery. Additional conduits included SVG, radial artery (RA), right internal thoracic artery (RITA), and gastroepiploic artery (GEA), according to target vessel characteristics and conduit availability.

EVH was performed in selected patients according to institutional practice.

### Angiographic assessment

Postoperative CAG was routinely performed before discharge or during early postoperative follow-up. Graft patency and stenosis severity were assessed by experienced cardiologists blinded to surgical strategy.

Early graft failure was defined as complete graft occlusion. SVG stenosis was categorized as <50%, 50%–90%, or ≥90% luminal narrowing. Stenosis at the anastomotic site was also recorded. Reintervention, including percutaneous coronary intervention (PCI) to native vessels or grafts, was documented.

### Endpoints

The primary endpoint was the incidence of GSV harvest-site wound complications, including leg wound dehiscence and surgical site infection. Secondary endpoints included early graft occlusion or significant SVG stenosis detected on postoperative CAG, reintervention related to SVG, and postoperative complications such as mediastinitis, reoperation for bleeding, atrial fibrillation, and neurologic events. Additional secondary outcomes were the length of intensive care unit (ICU) stay and total hospital stay, 30-day mortality, in-hospital mortality, and MACCE, defined as a composite of all-cause death, myocardial infarction, stroke, and repeat revascularization. Long-term SVG-related event-free survival and MACCE-free survival were also evaluated at 1, 3, 5, and 10 years.

### Follow-up

Clinical follow-up was conducted through outpatient visits and review of medical records. Survival and major adverse events were confirmed from hospital databases and direct patient contact when necessary.

### Statistical analysis

Continuous variables are presented as mean ± standard deviation (SD) or median with interquartile range (IQR), as appropriate. Categorical variables are expressed as frequencies and percentages.

Propensity scores were calculated using logistic regression modeling with pump usage as the dependent variable. Covariates included baseline patient characteristics (demographics and comorbidities) as well as operative risk profiles, including established surgical risk scores. One-to-one nearest-neighbor matching without replacement was performed.

Between-group comparisons were conducted using Student's t-test or the Mann–Whitney U test for continuous variables and the chi-square or Fisher's exact test for categorical variables. Survival curves for MACCE-free survival and SVG-related event-free survival were constructed using the Kaplan–Meier method and compared using the log-rank test. A two-sided *p*-value <0.05 was considered statistically significant. All statistical analyses were performed using JMP software, version 18.2.0 (SAS Institute Japan, Tokyo, Japan).

### Ethical considerations

The study protocol was approved by the institutional review board of our hospital, and the requirement for individual informed consent was waived due to the retrospective nature of the study. The study was conducted in accordance with the Declaration of Helsinki.

## Results

### Patient characteristics

**Figure 1:**
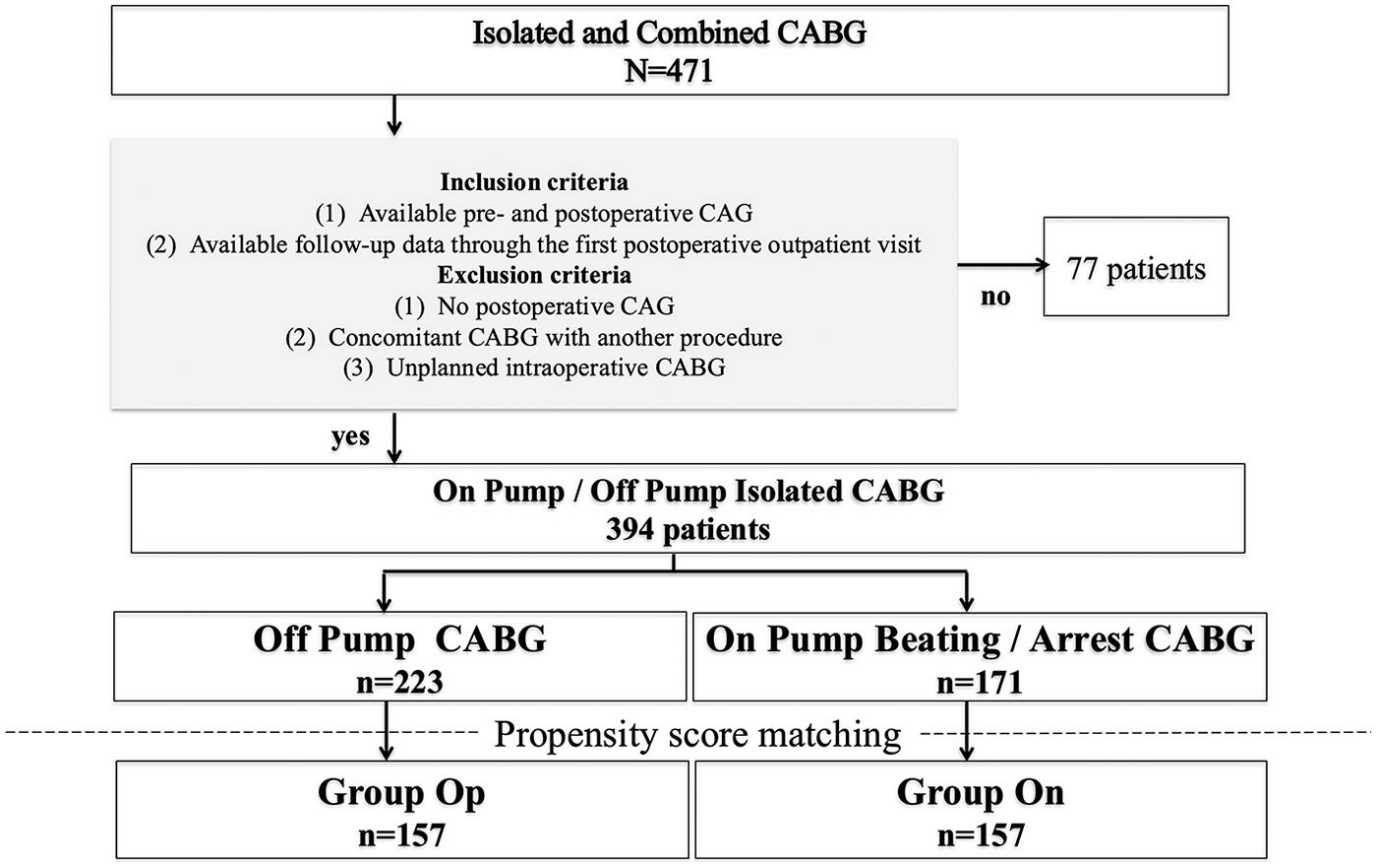
Study flow diagram. Among 471 patients who underwent isolated or combined coronary artery bypass grafting (CABG) during the study period, 394 met the inclusion criteria and were included in the analysis. **Inclusion criteria:** (1) availability of both preoperative and postoperative coronary angiography (CAG); and (2) availability of clinical follow-up data through at least the first postoperative outpatient visit. **Exclusion criteria:** (1) absence of postoperative CAG; (2) concomitant CABG performed with another major cardiac procedure; and (3) unplanned intraoperative CABG. Of the 394 eligible patients, 223 underwent off-pump CABG and 171 underwent on-pump CABG (including both beating-heart and arrested-heart procedures). Propensity score matching based on pump usage yielded 157 matched pairs: 157 patients in the off-pump group (Group Op) and 157 patients in the on-pump group (Group On). These matched cohorts were subsequently compared for perioperative, angiographic, and long-term clinical outcomes. CABG: coronary-artery bypass grafting, CAG: coronary angiography.

After propensity score matching, 157 patients were included in each group (On-pump CABG, *n* = 157; OPCAB, *n* = 157) (Figure 1). Baseline characteristics are summarized in Table 1. There were no significant differences between groups in age (68.1 ± 9.4 vs. 67.9 ± 9.1 years, *p* = 0.869), sex distribution, body mass index (BMI), prevalence of hypertension, hyperlipidemia, diabetes mellitus, smoking history, chronic kidney disease, hemodialysis, prior stroke, peripheral arterial disease, prior PCI, or EuroSCORE II. The proportion of patients with NYHA class III–IV symptoms was also comparable.

**Table 1:** Baseline patient characteristics (preoperative data).

Characteristic	On Pump CABG (*n* = 157)	OPCAB (*n* = 157)	*p*-value
Age, years	68.1 ± 9.4	67.9 ± 9.1	0.869
Male sex, *n* (%)	87.2	86.0	0.869
BMI, kg/m²	23.9 ± 4.2	23.1 ± 3.7	0.082
BMI ≥30, *n* (%)	6.6	3.3	0.195
Risk factors, *n* (%)			
Hypertension	80.7	80.9	1.00
Hyperlipidemia	68.2	62.5	0.337
Diabetes mellitus	45.5	49.3	0.567
Insulin use	11.9	14.8	0.397
Smoking history	70.5	66.0	0.549
Current smoker	13.9	13.3	1.00
Old myocardial infarction, *n* (%)	56.1	47.1	0.115
Family history, *n* (%)	23.9	15.6	0.194
Prior PCI, *n* (%)	22.2	23.7	0.788
Peripheral arterial disease, *n* (%)	7.0	8.9	0.678
CKD *n* (%)	9.0	12.7	0.364
Preoperative Max SCr, mg/dL, Mean ± SD	1.0 ± 0.9	1.0 ± 0.8	*0* *.* *938*
Hemodialysis, *n* (%)	3.8	5.1	0.786
Prior stroke, *n* (%)	7.7	9.6	0.688
NYHA class III–IV, *n* (%)	31.2	21.8	0.073
Left ventricular ejection fraction, %	53.0 ± 15.1	57.4 ± 15.5	0.015
Left main coronary artery stenosis, %	41.0	40.1	0.909
Number of diseased vessels ≥50%	2.7 ± 0.6	2.3 ± 0.8	<0.0001
Emergency/urgent surgery, *n* (%)	12.7	9.6	0.474
EuroSCORE II, median (IQR)	1.25 (0.69–2.25)	1.09 (0.76–2.18)	0.847
Follow-up duration, months, median (IQR)	23 (4–59)	28 (8.5–84)	0.018

Continuous variables are presented as mean ± standard deviation.

CABG, coronary artery bypass grafting; OPCAB, off pump coronary artery bypass grafting; BMI, body mass index; PCI, percutaneous coronary intervention; CKD, chronic kidney disease; NYHA, New York heart association; IQR, interquartile range.

Left ventricular ejection fraction (LVEF) was slightly lower in the On-pump group (53.0 ± 15.1% vs. 57.4 ± 15.5%, *p* = 0.015). The number of diseased vessels was greater in the On-pump group (2.7 ± 0.6 vs. 2.3 ± 0.8, *p* < 0.0001). Median follow-up duration was 23 (IQR 4–59) months in the On-pump group and 28 (IQR 8.5–84) months in the OPCAB group (*p* = 0.018).

### Operative data and graft strategy

Operative and graft-related data are presented in Tables 2, 3. The number of distal anastomoses was significantly higher in the On-pump group (2.9 ± 0.9 vs. 2.2 ± 1.0, *p* < 0.0001). Similarly, the number of SVG distal anastomoses was greater in the On-pump group (1.2 ± 0.8 vs. 0.7 ± 0.8, *p* < 0.0001). Regarding conduit selection, SVG use was more frequent in the On-pump group (81.5% vs. 51.6%, *p* < 0.0001), whereas radial artery (54.1% vs. 40.8%, *p* = 0.024) and gastroepiploic artery (1.9% vs. 8.9%, *p* = 0.011) were used more frequently in the On-pump and OPCAB groups, respectively. Use of the left internal thoracic artery was similar between groups. EVH was performed in approximately 27% of patients in both groups (27.4% vs. 28.7%, *p* = 0.900).

**Table 2:** Assessment of bypass graft anastomosis and wound complication.

Characteristic	On Pump CABG (*n* = 157)	OPCAB (*n* = 157)	*p*-value
Distal anastomoses, *n*, Mean ± SD	2.9 ± 0.9	2.2 ± 1.0	*<0* *.* *0001*
Distal anastomoses SVG used, n, Mean ± SD	1.2 ± 0.8	0.7 ± 0.8	*<0*.*0001*
Graft selection			
LITA, %	94.3	91.1	*0*.*387*
SVG, %	81.5	51.6	*<0*.*0001*
RITA, %	2.6	5.7	*0*.*257*
RA, %	54.1	40.8	*0*.*024*
GEA, %	1.9	8.9	*0*.*011*
Early results after graft harvest			
Graft occlusion early postoperative,%	9.6	10.3	*0*.*553*
Reintervention,%	9.6	7.1	*0*.*540*
Endoscopic saphenous vein harvesting,%	27.4	28.7	*0*.*900*
SVG occlusion early postoperative, %	5.7	4.5	*0*.*798*
Leg wound problems, %	1.3	0.6	*1*.*00*
Leg wound infection, %	0.6	0	*1*.*00*
SVG stenosis, <50%, %	0.6	0	*1*.*00*
SVG stenosis, 50–90%, %	1.3	3.2	*0*.*448*
SVG stenosis, ≥90%, %	0	0	*-*
Stenosis of SVG anastomosis, %	3.2	1.3	*0*.*448*
Reintervention to SVG, %	2.6	0	*0*.*123*

Leg wound problems were defined as delayed wound healing or local wound complications at the vein harvest site without clinical or microbiological evidence of infection.

Leg wound infection was defined as the presence of clinical signs of infection (e.g., purulent discharge, erythema, warmth), positive wound culture, and/or systemic findings such as fever or elevated inflammatory markers.

CABG, coronary artery bypass grafting; OPCAB, Off Pump coronary artery bypass grafting; SVG, saphenous vein graft; LITA, left internal thoracic artery; RITA, right internal thoracic artery; RA, radial artery; GEA, gastroepiploic artery.

**Table 3:** Clinical outcomes and complications according to surgical strategy (Off-pump vs. On-Pump CABG).

Result	On Pump CABG (*n* = 157)	OPCAB (*n* = 157)	*p*-value
Operation time, min, Mean ± SD	306 ± 84	235 ± 69	*<0*.*0001*
Cardiopulmonary bypass, min, Mean ± SD	130 ± 43	-	*-*
Aortic cross-clamp, min, Mean ± SD	81 ± 31	-	*-*
Cardioplegic arrest, *n* (%)	14.0	-	*-*
Converted to on-pump CABG, %	-	1.9	*-*
Required transfusion of red blood cells, %	64.3	49.7	*0*.*012*
Postoperative Max SCr, mg/dL, Mean ± SD	1.4 ± 1.2	1.2 ± 1.0	*0*.*443*
Neurologic dysfunction, %	0	0	*-*
Duration of mechanical ventilation (post operative days), median (IQR)	1 (1–1)	1 (0–1)	*0*.*063*
ICU stay (post operative days), Mean ± SD	5.0 ± 5.1	3.9 ± 2.5	*0*.*029*
Length of hospital stay.days, median (IQR)	20 (17–25)	17 (15–22)	*0*.*003*
Post operative atrial fibrillation,%	13.9	9.6	*0*.*283*
Reoperation for bleeding, %	1.9	0.6	*0*.*623*
Mediastinitis, %	3.3	0	*0*.*061*
30 days mortatlity, %	1.3	0.0	*0*.*498*
In-hospital deaths, %	1.9	0.0	*0*.*248*
MACCE, 12 months, %	12.1	12.1	*1,00*

CABG, coronary artery bypass grafting; OPCAB, off pump coronary artery bypass grafting; SD, standard deviation; SCr: serum creatinine; IQR, interquartile range; ICU, intensive care unit; MACCE, major adverse cardiac and cerebrovascular events.

### Angiographic outcomes

**Figure 2:**
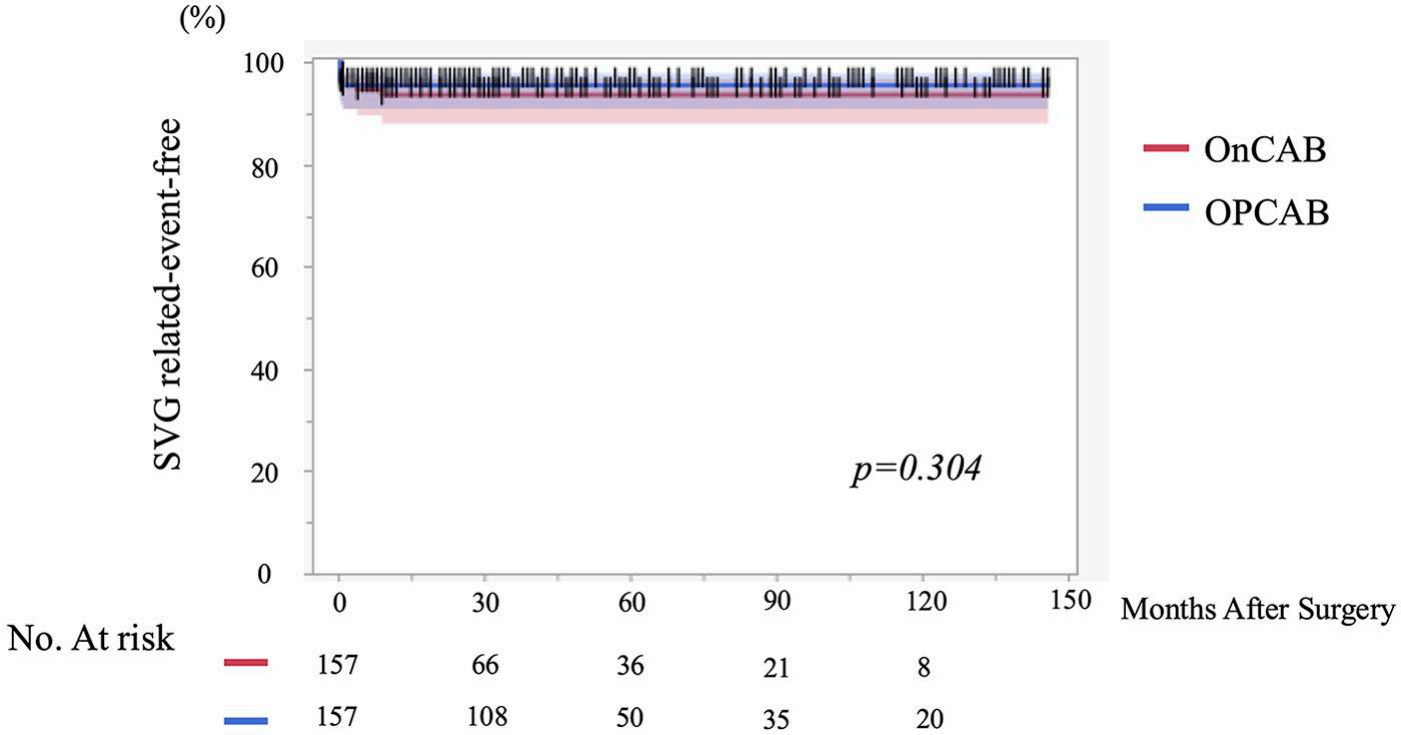
Early saphenous vein graft–related event–free survival according to surgical strategy (Off-pump vs. On-Pump CABG). Kaplan–Meier curves illustrating early postoperative saphenous vein graft (SVG)–related event–free survival in patients undergoing coronary artery bypass grafting (CABG) after propensity score matching. The matched cohort consisted of 157 patients in the off-pump group and 157 patients in the on-pump group. Follow-up was conducted for up to 140 months. Differences between groups were assessed using the log-rank test. SVG, saphenous vein graft; CABG, coronary artery bypass grafting.

Early postoperative CAG demonstrated no significant difference in overall graft occlusion rates between groups (9.6% vs. 10.3%, *p* = 0.553). Early SVG occlusion was also comparable (5.7% vs. 4.5%, *p* = 0.798). The distribution of SVG stenosis severity (<50%, 50%–90%, ≥90%) did not differ significantly between groups. Stenosis at the SVG anastomotic site (3.2% vs. 1.3%, *p* = 0.448) and reintervention to SVG (2.6% vs. 0%, *p* = 0.123) were likewise similar. SVG-related event-free survival at 1, 3, 5, and 10 years was 93.6%, 93.6%, 93.6%, and 93.6% in the On-pump group and 95.5%, 95.5%, 95.5%, and 95.5% in the OPCAB group, with no significant difference between groups (Figure 2).

### Harvest-Site and postoperative complications

The primary endpoint—leg wound complications related to GSV harvest—occurred infrequently and did not differ significantly between groups. Leg wound problems were observed in 1.3% of the On-pump group and 0.6% of the OPCAB group (*p* = 1.00). Leg wound infection occurred in 0.6% and 0%, respectively (*p* = 1.00). Mediastinitis occurred in 3.3% of On-pump patients and none of the OPCAB patients (*p* = 0.061). Reoperation for bleeding was comparable (1.9% vs. 0.6%, *p* = 0.623). Postoperative atrial fibrillation and neurologic dysfunction were also similar between groups.

### Perioperative course

Operation time was significantly longer in the On-pump group (306 ± 84 vs. 235 ± 69 min, *p* < 0.0001). Cardiopulmonary bypass time was 130 ± 43 min, and aortic cross-clamp time was 81 ± 31 min in the On-pump group.

Red blood cell transfusion was more frequently required in the On-pump group (64.3% vs. 49.7%, *p* = 0.012). ICU stay was longer in the On-pump group (5.0 ± 5.1 vs. 3.9 ± 2.5 days, *p* = 0.029), as was total hospital stay (median 20 vs. 17 days, *p* = 0.003).

### Mortality and long-term outcomes

Thirty-day mortality was 1.3% in the On-pump group and 0% in the OPCAB group (*p* = 0.498). In-hospital mortality was 1.9% and 0%, respectively (*p* = 0.248), without statistical significance.

**Figure 3:**
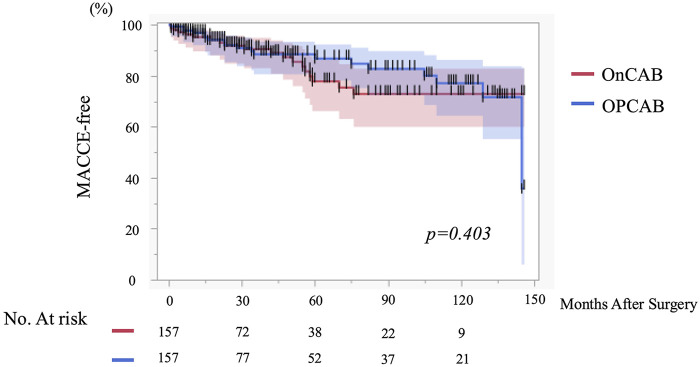
Short- to midterm MACCE-free survival after CABG according to surgical strategy (Off-pump vs. On-Pump CABG). Kaplan–Meier curves showing short- to midterm major adverse cardiac and cerebrovascular event (MACCE)–free survival after coronary artery bypass grafting (CABG) in the propensity score–matched cohort. Patients were stratified into off-pump and on-pump groups. MACCE was defined as a composite endpoint of all-cause mortality, myocardial infarction, stroke, or repeat revascularization. Given the retrospective study design and the absence of protocol-mandated long-term angiographic follow-up, this analysis primarily reflects early and intermediate clinical outcomes rather than late graft-related events. Differences between groups were evaluated using the log-rank test. MACCE, major adverse cardiac and cerebrovascular events; CABG, coronary artery bypass grafting.

MACCE at 12 months occurred in 12.1% of patients in both groups (*p* = 1.00). Long-term MACCE-free survival at 1, 3, 5, and 10 years was 95.2%, 91.8%, 77.8%, and 72.9% in the On-pump group and 96.9%, 88.5%, 86.8%, and 77.1% in the OPCAB group, respectively, with no significant difference between groups (Figure 3).

## Discussion

In this propensity score–matched single-center study conducted in the contemporary minimally invasive era, the principal finding was that GSV harvest-site wound complications were infrequent and did not differ significantly between OPCAB and on-pump CABG. This low event rate suggests that, in current clinical practice, harvest-site morbidity has become uncommon and may no longer be strongly influenced by pump strategy alone. The principal findings were: (1) harvest-site wound complications were infrequent and comparable between groups; (2) early graft occlusion and SVG stenosis rates did not differ significantly; and (3) long-term MACCE-free survival was similar, although OPCAB was associated with shorter operative time, reduced transfusion requirements, and shorter hospitalization.

The low incidence of wound complications observed in this study likely reflects multiple factors. First, advances in perioperative management and infection control may have contributed to improved wound healing. Second, EVH, which was used in approximately one-quarter of patients in both groups, has been associated with reduced surgical site complications compared with open harvesting techniques. Third, patient selection and optimized perioperative care may have further mitigated wound-related risk.

Taken together, these findings suggest that contemporary improvements in surgical technique and perioperative management may outweigh any potential impact of cardiopulmonary bypass avoidance on wound outcomes.

### Harvest-Site morbidity in the contemporary Era

Avoidance of cardiopulmonary bypass (CPB) has historically been associated with attenuation of systemic inflammatory response and early postoperative morbidity (2). However, in the present cohort, leg wound complications were rare and not influenced by pump usage. These findings suggest that, in current practice, conduit harvesting strategy and perioperative management may be more influential determinants of wound outcomes than CPB avoidance itself.

Endoscopic vein harvesting (EVH) was used in approximately one-quarter of patients in both groups. EVH has been shown to reduce leg wound infection and dehiscence compared with open techniques (8, 9, 14). Although earlier analyses raised concerns regarding graft patency (15), subsequent long-term data have demonstrated no significant difference in clinical outcomes (16, 17). Balanced EVH utilization in our study likely contributed to the uniformly low incidence of harvest-site morbidity.

Importantly, our institution has previously reported favorable wound outcomes and graft performance in contemporary CABG practice, including minimally invasive and conduit-strategy refinements (18, 19). The present analysis extends those findings by demonstrating that pump strategy alone does not appear to independently influence GSV harvest-site complications within a standardized institutional framework.

### Early angiographic graft integrity

In our matched cohort, early postoperative CAG demonstrated no significant difference in overall graft occlusion or SVG stenosis severity. Although the on-pump group underwent a greater number of distal and SVG anastomoses, graft integrity was similar. These findings align with contemporary evidence suggesting that surgical expertise and case selection are critical determinants of OPCAB outcomes (20, 21). The observed graft occlusion rates in this study may appear relatively high compared with those reported in some prior studies. However, this finding should be interpreted in the context of routine postoperative coronary angiography performed in all patients, regardless of clinical symptoms. Such systematic assessment enables the detection of subclinical graft failure that may not be captured in studies relying solely on symptom-driven or clinically indicated angiographic evaluation. Therefore, the reported rates likely reflect a more comprehensive and objective assessment of early graft integrity rather than inferior surgical performance.

Furthermore, our previous institutional analyses have emphasized the importance of objective angiographic assessment in understanding graft durability and clinical events (18, 22). The present data corroborate those observations by demonstrating comparable early angiographic performance between pump strategies. Although the primary focus of this study was harvest-site morbidity, we additionally evaluated early angiographic graft integrity and long-term clinical outcomes to provide complementary mechanistic and clinical context. Importantly, no significant differences were observed in graft occlusion, SVG stenosis, or MACCE between the two strategies, supporting the overall equivalence of OPCAB and on-pump CABG in this cohort.

Although the primary focus of this study was harvest-site morbidity, we additionally evaluated early angiographic graft integrity and long-term clinical outcomes to provide complementary mechanistic and clinical context. Importantly, no significant differences were observed in graft occlusion, SVG stenosis, or MACCE between the two strategies, supporting the overall equivalence of OPCAB and on-pump CABG in this cohort.

### Perioperative course and resource utilization

OPCAB was associated with shorter operative duration, lower transfusion requirements, and reduced ICU and hospital stay. These findings are consistent with prior meta-analyses demonstrating reduced blood product utilization and shorter recovery with off-pump techniques (20, 21). CPB-related hemodilution, platelet dysfunction, and inflammatory activation may partly explain these differences (2).

While it may be anticipated that pump strategy alone would have a limited impact on peripheral wound healing, OPCAB has been associated with reduced systemic inflammatory response and avoidance of cardiopulmonary bypass, providing a physiological rationale for investigation (23). However, our findings suggest that such theoretical advantages do not translate into clinically meaningful differences in harvest-site outcomes in the contemporary setting.

In the present study, operative and cardiopulmonary bypass times in the on-pump group may appear relatively prolonged. This likely reflects differences in case complexity, including a greater number of distal anastomoses and a higher prevalence of multivessel disease in this group. In addition, institutional practice patterns and the inclusion of complex or comprehensive revascularization strategies may have contributed to longer operative durations. Despite these factors, no significant differences were observed in major clinical outcomes, suggesting that the overall procedural safety was maintained.

Although mortality and major complications were similar, the reduction in perioperative resource utilization may have implications for enhanced recovery pathways and institutional efficiency.

### Long-Term clinical outcomes

Long-term MACCE-free survival did not differ significantly up to 10 years. These findings are consistent with pooled analyses showing equivalent long-term survival between on-pump and off-pump CABG when performed by experienced surgeons (11).

Despite differences in conduit selection—with greater SVG use in the on-pump group—clinical outcomes remained comparable. The durability of arterial grafts has been highlighted in prior literature (24), yet our results suggest that overall revascularization strategy and technical execution may outweigh pump status in determining long-term prognosis.

### Limitations

Several important limitations warrant consideration.

First, surgical strategy was determined by the attending surgeon rather than randomized allocation. Therefore, selection bias cannot be excluded. The on-pump group exhibited a higher number of diseased vessels and distal anastomoses, suggesting greater anatomical complexity. Surgeon preference may have influenced both pump selection and revascularization completeness. Despite propensity score matching, residual confounding related to case severity and operative complexity likely remains.

Second, although early postoperative CAG was incorporated, long-term angiographic follow-up was not routinely performed. CAG during follow-up was generally triggered by clinical events or ischemic suspicion. Consequently, subclinical SVG stenosis or silent graft failure may not have been detected. Our evaluation of SVG-related outcomes therefore likely underestimates the true incidence of late graft degeneration.

Third, this was a retrospective single-center study spanning more than a decade, during which operative strategy and institutional experience evolved. Temporal trends may have influenced results.

Finally, the low incidence of the primary endpoint limits the statistical power to detect small differences between groups. Therefore, these findings should be interpreted with caution, and larger multicenter studies may be required to confirm these observations.

## Conclusions

Within a contemporary minimally invasive framework, OPCAB did not significantly reduce GSV harvest-site wound complications compared with on-pump CABG. Early graft integrity and long-term MACCE-free survival were comparable. While OPCAB was associated with improved perioperative resource utilization, overall clinical outcomes were similar. These findings suggest that, in experienced centers, conduit harvesting strategy, technical expertise, and patient selection may exert greater influence on outcomes than pump avoidance alone.

## Data Availability

The raw data supporting the conclusions of this article will be made available by the authors upon reasonable request, in accordance with institutional and ethical regulations, and with appropriate safeguards for patient confidentiality.
